# Skin microbiota: pathogenic roles and implications in atopic dermatitis

**DOI:** 10.3389/fcimb.2024.1518811

**Published:** 2025-01-14

**Authors:** Cong Huang, Fan Zhuo, Yang Guo, Siyu Wang, Kaoyuan Zhang, Xiahong Li, Wenkui Dai, Xia Dou, Bo Yu

**Affiliations:** ^1^ Department of Dermatology, Skin Research Institute of Peking University Shenzhen Hospital, Peking University Shenzhen Hospital, Shenzhen, China; ^2^ Shenzhen Key Laboratory for Translational Medicine of Dermatology, Shenzhen Peking University - the Hong Kong University of Science and Technology Medical Center, Shenzhen, China; ^3^ Department of Epidemiology and Statistics, School of Public Health, Hebei Key Laboratory of Environment and Human Health, Hebei Medical University, Shijiazhuang, Hebei, China; ^4^ Department of Dermatology, Peking University Shenzhen Hospital, Shenzhen, China; ^5^ Department of Dermatology, Peking University Shenzhen Hospital, Hubei University of Medicine, Shiyan, Hubei, China; ^6^ Department of Obstetrics and Gynecology, Peking University Shenzhen Hospital, Shenzhen, China

**Keywords:** atopic dermatitis, skin microbiota, *Staphylococcus aureus*, pathogenesis, microbiota-based therapy

## Abstract

Atopic dermatitis (AD) is a chronic and inflammatory skin disorder characterized by impaired barrier function and imbalanced immunity. Recent advances have revealed that dysbiosis of skin microbiota plays important roles in the pathogenesis and development of AD. Meanwhile, endogenous and external factors contribute to the dysbiosis of skin microbiota in AD. Additionally, various treatments, including topical treatments, phototherapy, and systemic biologics, have demonstrated positive impacts on the clinical outcomes, alongside with the modulations of cutaneous microbiota in AD patients. Importantly, therapeutics or products regulating skin microbiota homeostasis have demonstrated potential for AD treatment in early clinical studies. In this review, we underline changes of the skin microbiota correlated with AD. Meanwhile, we provide an overview of the skin microbiota regarding its roles in the pathogenesis and development of AD. Finally, we summarize therapeutic strategies restoring the skin microbial homeostasis in AD management.

## 1 Introduction

Atopic dermatitis (AD) is a long-lasting and inflammatory skin disease. It usually appears during infancy and childhood; however, people of all ages can get this skin condition (Wollenberg et al., 2016; Weidinger et al., 2018). Symptoms of AD range from excessively dry, extremely itchy skin to painful skin, which can flare in multiple areas of the body sites, severely affecting the life quality of patients worldwide (Sidbury et al., 2014; Silverberg et al., 2015; Vakharia et al., 2017; Silverberg et al., 2019). Although it is clearly that genetics, immune system, and environmental factors play roles in AD pathogenesis, the precise mechanism that causes atopic dermatitis remains elusive (Weidinger and Novak, 2016; Weidinger et al., 2018; Langan et al., 2020). Recently, increasing evidence revealed that dysbiosis of skin microbiota contributes to AD development (Williams and Gallo, 2015; Bjerre et al., 2017; Tao et al., 2022).

The skin microbiota are mainly composed of numerous bacterial species, as well as fungi and virus (Flowers and Grice, 2020; Whiting et al., 2024). Homeostasis of the skin microbiota benefits the host by providing support to the skin barrier function and inhibiting pathogen colonization (Prescott et al., 2017; Parlet et al., 2019; Swaney and Kalan, 2021; Hammond et al., 2022). Meanwhile, the skin microbiota can modulate the host innate and adaptive immunity (Coates et al., 2019; Paller et al., 2019; Boxberger et al., 2021), both of which are core components of the skin immune system (Richmond and Harris, 2014; Coates et al., 2018; Ong, 2022). Thus, the skin microbiota homeostasis is apparently important in maintaining healthy skin, while its dysbiosis contributes to skin pathology.

## 2 The regulatory roles of skin microbiota in AD pathogenesis

**Figure 1:**
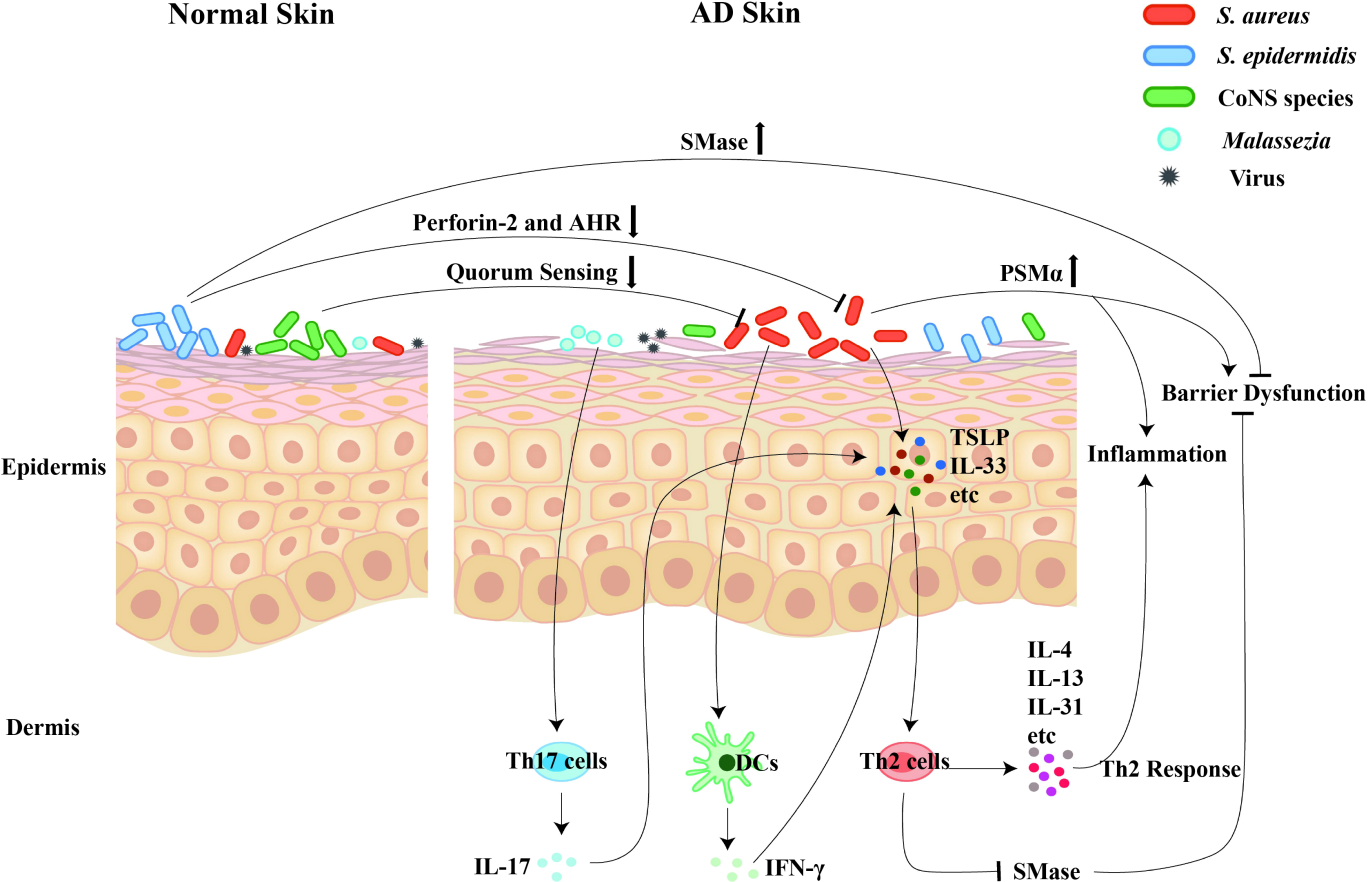
The regulatory roles of cutaneous microbiota on the pathogenesis and development of AD. Dysbiosis of the skin microbiota contributes to the pathogenesis and development of AD. Firstly, the *S. aureus* colonization promotes the disease progression through inducing the skin inflammation, triggering the skin barrier damage, and mediating the Th2 immune response. Secondly, the commensals, including the *S. epidermidis* and the CoNS species, contribute to skin homeostasis via inhibiting the *S. aureus* overgrowth and maintaining the skin’s barrier function. Thirdly, the commensal fungi, especially the over-presentation of *Malassezia*, selectively induce Th17 activation and subsequent skin inflammation.

Given that skin microbiota dysbiosis is increasingly implicated as a contributor to the pathogenesis of AD, the effects and molecular mechanisms of cutaneous microbiota on the development of AD cannot be overlooked (**Figure 1**).

### 2.1 Staphylococcus aureus (S. aureus) plays crucial roles in AD pathogenesis

*S. aureus* on the skin is positively correlated with disease severity and represents one of the main triggers for the worsening of skin lesions in AD patients (Tauber et al., 2016; Totté et al., 2016). In the mouse model of AD, epicutaneous exposure of skin to *S. aureus* induces inflammation (Liu et al., 2017). Mechanistically, phenol-soluble modulin α (PSMα) secreted by *S. aureus* contributes to skin inflammation (Liu et al., 2017). Additionally, *S. aureus*-expressed PSMα induces release of pro-inflammatory cytokines/chemokines from keratinocytes, as well as subsequent IL-17 production from immune cells, leading to cutaneous inflammation in a murine infection model (Syed et al., 2015; Nakagawa et al., 2017). Consistently, Williams et al. reported that the proteases and PSMα secreted by *S. aureus* lead to epidermal proteolysis and skin barrier damage (Williams et al., 2019), further confirming that *S. aureus* contributes to AD pathogenesis.

Upon incubation with keratinocytes, heat killed *S. aureus* from AD patients are strongly agglutinated inside the cytoplasm, where they are located in lysosomes and promote the secretion of IL-1α (Moriwaki et al., 2019). In addition, the inoculation with *S. aureus* increased IL-1β and IL-18 production, whereas silencing of NLRP1 decreased the secretion of these cytokines in keratinocytes, suggesting that *S. aureus* may contribute to the pathogenesis of AD through NLRP1 inflammasome/IL-1β and IL-18 axis (Vaher et al., 2023). Moreover, *S. aureus* enhances the release of thymic stromal lymphopoietin (TSLP) in human keratinocytes, and mediates the Th2-type inflammation (Vu et al., 2010), providing further clue for the association between *S. aureus* colonization and AD disease progression.

Using shotgun metagenomic sequence analysis, Byrd et al. revealed that AD flares exhibited greater *S. aureus* predominance in patients with severe disease (Byrd et al., 2017). Specifically, *S. aureus* from AD patients with more severe flares induced epidermal thickening and expansion of cutaneous Th2 and Th17 cells in the murine models (Byrd et al., 2017), indicating its contributory role to AD development.

*S. aureus* is unique among staphylococcal species, as it’s able to induce rapid release of IL-33 from keratinocytes, identifying it as a dominant type 2 immunity trigger underlying AD pathogenesis (Al Kindi et al., 2021). Interestingly, Th2 cytokine exposure increases the sensitivity to *S. aureus* alpha toxin-induced cell lethality in keratinocytes, which is more commonly observed in skins from AD patients compared with healthy individuals (Brauweiler et al., 2014). Furthermore, Th2 cytokines decrease levels of sphingomyelinase (SMase) and production of lamellar bodies, which are critical for cleaving alpha toxin receptor and keeping epidermal barrier formation, respectively. Finally, SMase prevents Th2-mediated cell death (Brauweiler et al., 2014), uncovering a mechanism that Th2 cytokine exacerbating *S. aureus*-induced AD pathogenesis.

In fact, in addition to *S. aureus* alpha toxin, several other staphylococcal exotoxins also play crucial roles in the pathogenesis of AD. Nakamura et al. found that δ-toxin produced by *S. aureus* potently facilitated the degranulation of mast cells (Nakamura et al., 2013). Further studies demonstrated that the skin colonization by *S. aureus* enhanced the production of immunoglobulin-E (IgE) and IL-4, along with skin inflammation, in a δ-toxin-dependent manner. Interestingly, IgE could amplify the δ-toxin-induced mast cell degranulation in the absence of antigens. Moreover, mast cell deficiency and subsequent reconstitution could either abrogate or restore the production of IgE induced by δ-toxin, respectively, in a dermatitis mouse model. Clinically, *S. aureus* isolated from AD patients produced large amounts of δ-toxin (Nakamura et al., 2013). Therefore, the *S. aureus*/δ-toxin/IgE/mast cell axis represents a novel mechanism in the pathogenesis of AD.

Strikingly, the skin inflammation leads to a rapid recruitment of neutrophils, which correlates with enhanced *S. aureus* colonization. On the contrary, depletion of neutrophils reduces the skin colonization of *S. aureus* (Bitschar et al., 2020). Meanwhile, the interaction of neutrophil extracellular traps (NETs, released by infiltrating neutrophils) with keratinocytes are responsible for increased *S. aureus* colonization (Bitschar et al., 2020), suggesting that inflammatory environment contributes to enhanced *S. aureus* colonization in AD pathogenesis.

Moreover, monocyte-derived Langerhans cells stimulated by AD associated-*S. aureus* induce rapid proliferation of T cells (Iwamoto et al., 2017), demonstrating that *S. aureus* can skew T cell responses in AD pathogenesis. Additionally, exposure of *S. aureus* secretomes to monocyte-derived dendritic cells (moDC) promotes the release of pro-inflammatory IFN-γ (Laborel-Préneron et al., 2015). Meanwhile, allogeneic moDC exposed to *S. aureus* secretome induces CD4^+^ T cell proliferation. Furthermore, the *S. aureus* secretome inhibits Treg activity (Laborel-Préneron 9et al., 2015). Therefore, colonization of *S. aureus* on AD skins regulates cutaneous inflammation through multiple different pathways.

### 2.2 Roles of commensal microbiota in AD pathogenesis

*S. aureus* is known to exacerbate AD, whereas *Staphylococcus epidermidis* (*S. epidermidis*) has been considered as a beneficial commensal organism. Interestingly, *S. epidermidis* activates and upregulates Perforin-2 (P-2), while the upregulation of P-2 correlates with increased killing of intracellular *S. aureus* in skin cells, thereby protecting the host from skin infections (Strbo et al., 2019; Pastar et al., 2020). Moreover, *S. epidermidis* contributes to skin’s physical integrity by secreting SMase (Zheng et al., 2022). In mouse models, *S. epidermidis* increases the skin ceramide levels and prevents skin water loss in a SMase-dependent way (Zheng et al., 2022), demonstrating its crucial role in maintaining the skin’s barrier function. *S. epidermidis* also plays important roles in keeping the proper differentiation and repairment of the epidermal barrier, which are partially mediated by aryl hydrocarbon receptor (AHR) signaling in keratinocytes (Uberoi et al., 2021). Interestingly, *S. epidermidis* strains produce strong cysteine protease activity when grow at high density, which is able to degrade desmoglein-1 and LL-37 *in vitro*, and disrupt the physical barrier and induce skin inflammation *in vivo* (Cau et al., 2021). Clinically, the abundance of *S. epidermidis* is increased on the skin of some patients with AD, which is correlated with disease severity (Cau et al., 2021). Mechanistically, phenol-soluble modulin (PSM) peptides produced by *S. epidermidis* induce host defense and are cytotoxic to human keratinocytes. Moreover, the expression of PSMδ from *S. epidermidis* is positively correlated with disease severity in AD patients (Williams et al., 2023). Thus, the over-presence of *S. epidermidis* found on some AD patients can act similarly to *S. aureus* and contribute to AD pathogenesis.

As discussed in “2.1 Section“, *S. aureus* secretes PSMα and contributes to skin inflammation and barrier dysfunction. Interestingly, coagulase-negative staphylococci (CoNS) species residing on normal skin, inhibit *S. aureus* growth and subsequently lead to decreased PSMα expression (Williams et al., 2019), showing a concept that normal skin microbiota contributes to epithelial barrier homeostasis. Meanwhile, the CoNS strains with antimicrobial activity are common on the healthy skins but rare on the AD skins. Moreover, application of these CoNS strains to mice or reintroduction of them to AD patients confirms their defense against *S. aureus* (Nakatsuji et al., 2017). Further, spent media from the CoNS species can inhibit quorum sensing by *S. aureus* (Paharik et al., 2017). Specifically, the autoinducing peptide of CoNS species is responsible for the reduction of quorum sensing, and it dramatically reduces the cutaneous bacterial burden in a murine model of infection (Paharik et al., 2017). Thus, the skin commensals protect against skin dysbiosis, especially the *S. aureus* outgrowth, and impact disease outcomes in AD.

Commensal fungi of the skin, such as those of the genus *Malassezia*, are also associated with AD pathogenesis. For instance, the presence of *Malassezia*, selectively induce IL-17 and related cytokines, leading to aggravated cutaneous inflammation. Moreover, AD patients show enhanced frequency of Th17 subsets than healthy individuals, which is *Malassezia* involved and specific (Sparber et al., 2019). Thus, *Malassezia*-induced Th17 response is pivotal in promoting skin inflammation.

The most common sebaceous skin commensal yeasts are *Malassezia*, while the dominant secreted Malassezia globosa protease is Malassezia globosa Secreted Aspartyl Protease 1 (MGSAP1), which can rapidly hydrolyze Staphylococcus aureus protein A and holds anti-biofilm properties against *S. aureus* (Li et al., 2018), indicating that *Malassezia* and its enzyme maybe beneficial for skin health. In AD patients, upregulated MGSAP1 is observed in lesional skins, as compared to healthy skins (Goh et al., 2022). Functional loss of MFSAP1 leads to reductions in the cell adhesion and dispersal in both cultured and a human 3D epidermis models. Furthermore, MGSAP1 contributes to inflammation in a murine model of *Malassezia* colonization (Goh et al., 2022). Thus, MGSAP1 plays crucial roles in enabling fungi (*Malassezia*) colonization and promoting the skin barrier disruption.

### 2.3 The microbiota-host interaction contributes to AD pathogenesis

The overabundant colonization by *S. aureus* may trigger the aggravation of AD skins. Meanwhile, the initiation and progression of AD may require the adherence of *S. aureus* to the skin, the mechanism of which remains largely unknown. Interestingly, fibronectin and fibrinogen enhance the binding of *S. aureus* to the skins of AD patients (Cho et al., 2001). Meanwhile, the *S. aureus* itself stimulates keratinocytes to increase their trypsin activity, as well as degradation of desmoglein-1 and filaggrin (Williams et al., 2017), illustrating that *S. aureus* influences the skin barrier integrity by stimulating endogenous proteolytic activity.

Adhesion of *S. aureus* to corneocytes in the stratum corneum is also a key initial event in colonization. It was recently reported that *S. aureus* interacted with and took advantage of the host protein corneodesmosin, to facilitate its binding with AD corneocytes (Towell et al., 2021). The abnormalities of epidermal barrier in AD can also alter the entry of *S. aureus* into the dermis, while the dermal dysbiosis results in increased inflammation and exacerbated disease severity (Nakatsuji et al., 2016), defining a novel mechanism by which *S. aureus* contributes to AD development. Keratinocytes lacking JunB exhibit higher MyD88 levels, which promotes the colonization of *S. aureus* (Uluçkan et al., 2019). Additionally, the spontaneous *S. aureus* colonization in JunB deficient mice shows a large transcriptomic overlap with AD (Uluçkan et al., 2019). Thus, JunB works as an upstream regulator of microbiota-immune interaction in AD pathogenesis.

### 2.4 Roles of microbial metabolites in AD pathogenesis

The metabolites of skin microbiota can also act as a regulator of AD inflammation (Li and Yosipovitch, 2020). For instance, indole-3-aldehyde (IAId), a skin microbiota-derived tryptophan metabolite, is significantly lower in the lesional skins of AD patients than that of healthy individuals (Yu et al., 2019). Moreover, IAId attenuates the skin inflammation in MC903-induced mice through inhibiting TSLP expression in keratinocytes (Yu et al., 2019), indicating a novel mechanism that skin microbial metabolites modulating AD pathogenesis. In the Langerhans cells (LCs), IAId increases the production of indoleamine 2,3-dioxygenase and IL-10 (Liu et al., 2020). Additionally, IAId induces a mature phenotype of LCs, leading to the inhibition of CD4^+^ T cell proliferation and IL-10 secretion (Liu et al., 2020), revealing its negative regulation of LC function in AD pathogenesis.

AD patients also demonstrates a dysregulated lipidome of sebum and aberrant lipid metabolism in sebaceous glands (Yin et al., 2023). Interestingly, the levels of sebum and its microbial metabolite, propionate, are lower on the skins of AD patients compared with those of healthy individuals (Qiu et al., 2022). Mice lacking sebum spontaneously develop AD-like dermatitis, which can be improved by topical propionate application (Qiu et al., 2022), pointing the sebum-propionate axis as a protector pattern in AD. A proof-of-concept clinical study further demonstrates the beneficial of topical propionate application in AD patients (Qiu et al., 2022), highlighting a possible therapeutic for AD treatment.

## 3 Endogenous factors that contribute to the dysbiosis of skin microbiota in AD patients

**Figure 2:**
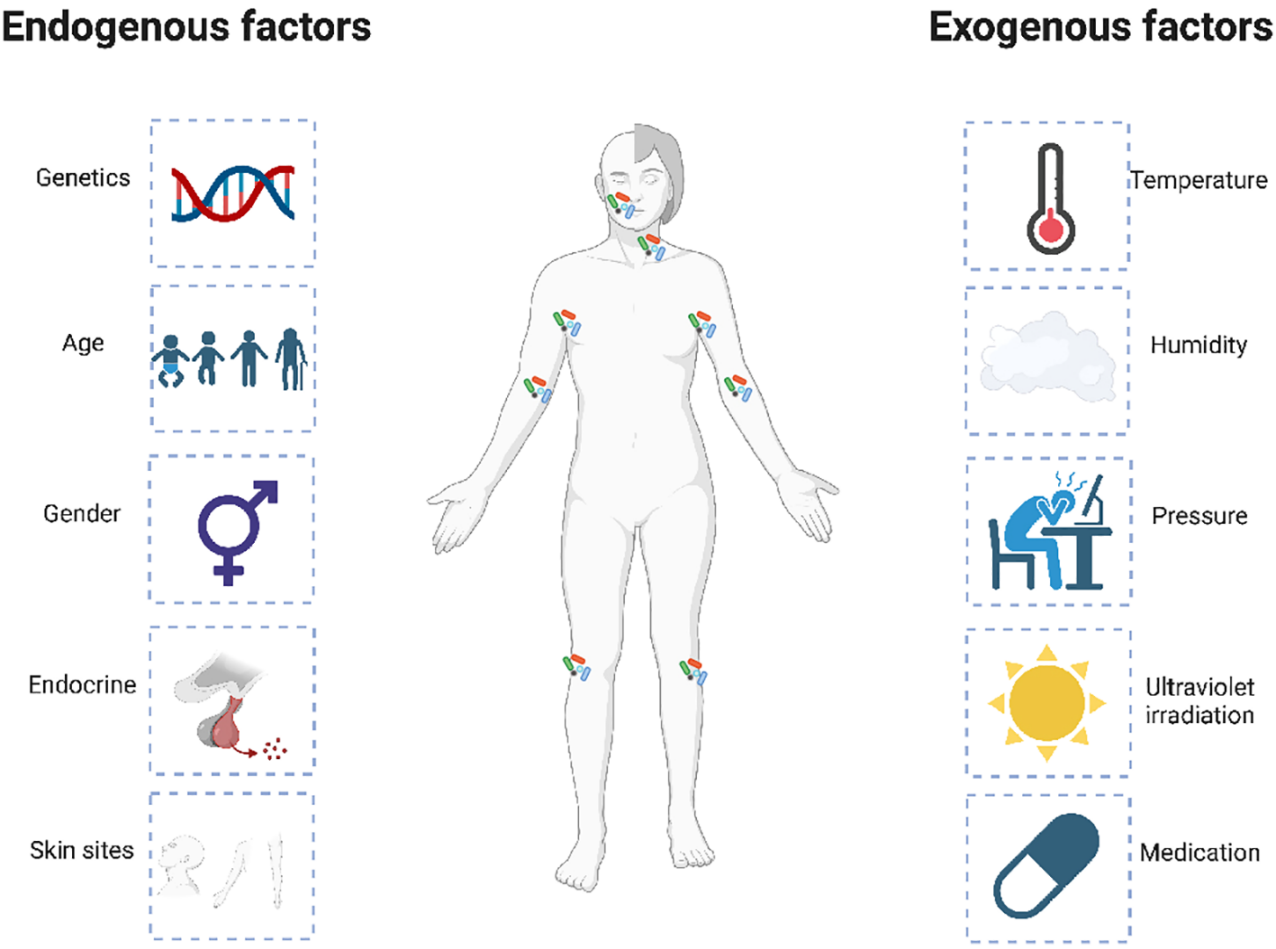
Endogenous and exogenous factors that influence the dysbiosis of skin microbiota in AD patients. The distinct microbiotal colonization on the AD skins is affected by multiple endogenous and exogenous factors. The endogenous factors are mainly composed of genetics, age, and skin sites, etc, while the exogenous factors are mainly composed of temperature, humidity, pressure, ultraviolet irradiation, and especially, various treatments.

Accumulating clues have demonstrated that microbial dysbiosis can result in inflammatory skin diseases, such as atopic dermatitis (Williams and Gallo, 2015; Bjerre et al., 2017; Tao et al., 2022). The affected individuals are characteristically prone to colonized by different microorganisms, which may reflect the disease condition of patients. Specifically, the skin microbiota of AD patients shows increased *Staphylococcus aureus* (*S. aureus*) load, which is correlated with reduced bacterial diversity (Francuzik et al., 2018; Hui et al., 2020; Poh et al., 2022). Additionally, increased *S. aureus* abundance is positively related to exacerbated disease severity in AD (Edslev et al., 2021). Moreover, the intra-host genetic heterogeneity of the colonizing *S. aureus* provides evidence for within-host selection in AD patients (Harkins et al., 2018). In general, the distinct microbiotal colonization on AD skins are affected by endogenous and external factors (Shi et al., 2016). The endogenous factors are mainly composed of genetics, age, and skin sites, etc, while the exogenous factors are mainly composed of temperature, humidity, pressure, ultraviolet irradiation, and especially, various treatments (**Figure 2**).

### 3.1 Genetic variation (filaggrin gene mutation) affects the microbial heterogeneity in AD

It is widely accepted that the genetic variations are closely associated with AD that shows high heritability. One of the main genes related to AD is the Filaggrin gene (FLG), the null mutation of which is a major risk factor for skin barrier dysfunction and tightly related to AD pathogenesis (Palmer et al., 2006; Margolis et al., 2012; Margolis et al., 2019; Hoyer et al., 2022). Interestingly, FLG mutation correlates with disease severity, as well as the skin microbiotal alteration in AD patients (Baurecht et al., 2018; Clausen et al., 2018; van Mierlo et al., 2022).

In pediatric patients with difficult-to-treat AD, the prevalence of FLG mutation is around 40%. Meanwhile, a significant effect of the FLG mutation on the overall skin microbiome is observed (van Mierlo et al., 2022). In adults, altered skin microbiota in AD patients is also detected, with microbial composition linked to the FLG mutation (Clausen et al., 2018). Importantly, the alpha diversity of microbiome shows inversely correlation with disease severity, and is lower in AD patients compared with healthy individuals (Clausen et al., 2018), proposing a possible correlation between skin microbiota and host genetics. Coincidentally, Baurecht et al. reported that AD displayed a distinct community structure, with the FLG-deficient skins showed a microbiota composition resembling the AD-related pattern (Baurecht et al., 2018). By incorporating the host genome and skin microbial composition into a computational model, Barnes et al. found that the bacterial communities varied markedly between AD patients without and with FLG mutations, further confirming the association between FLG mutation and the skin microbiotal diversity in AD (Barnes et al., 2022).

In Indian AD patients, the prevalence of FLG missense variants correlated with AD are significantly less than those reported in Europeans (Nath et al., 2020). Meanwhile, damaging missense single nucleotide polymorphisms of FLG gene are associated with the relative abundance of bacteria species (Nath et al., 2020). Specifically, increased *S. aureus* abundance in AD patients with FLG mutation is observed, suggesting that genetic differences are important for bacterial colonization on AD skins (Clausen et al., 2017). Mechanistically, FLG expression protects keratinocyte from staphylococcal alpha-toxin-induced death (Brauweiler et al., 2013). This partially explain why *S. aureus* α-toxin preferentially targets and destroys FLG-deficient keratinocytes, which also provides a mechanism for *S. aureus*-mediated exacerbation in AD pathogenesis.

### 3.2 Impact of age on the skin microbiota in AD patients

The skin microbiomes differ significantly between children and adults, suggesting the possible role of age in altering the skin microbiota composition (Dominguez-Bello et al., 2010; Capone et al., 2011). For instance, Kennedy et al. demonstrated that skin microbiome, including the bacterial community and diversity, shift over time in infants (Kennedy et al., 2017). Interestingly, infantile AD patients do not have noticeably dysbiotic communities before disease flares and were not colonized by *S. aureus* before having AD. Moreover, reduced skin microbiome diversity is associated with an increased risk of AD in infancy (Halling et al., 2023). Additionally, commensal *Staphylococci* are less colonized in infants affected at month 12, suggesting their protective role against AD development (Kennedy et al., 2017). However, further studies are needed to confirm these observations.

In adult patients with AD, cutaneous *S. aureus* colonization positively correlates with skin barrier impairment, as well as the subsequent sensitization to antigens of skin-associated microorganisms (Jinnestål et al., 2014), illustrating its importance in eczema pathogenesis. Besides, *S. aureus* also play important roles in the pathophysiology of childhood AD (Hon et al., 2016). In infancy, *S. aureus* is more prevalent on the skin of infants who developed AD later on (Meylan et al., 2017). Further, infants positive for cutaneous *S. aureus* colonization are younger than uncolonized infants at AD onset (Meylan et al., 2017), suggesting that *S. aureus* colonization contributes to clinical AD onset at an early age, even in infancy.

In a large cohort study, Shi et al. systematically compared the skin microbiomes among different age groups to determine the effects of human physical developmental stages on AD (Shi et al., 2016). In the skin microbiome of healthy individuals, young children (age 2-12) demonstrated more diverse than adults (age 18-62), with distinct beta diversity and significant differences at both genus and specie levels. Similarly, differences between young children and adults and/or teenagers (age 13-17) were observed in the skin microbiome of AD patients (Shi et al., 2016). Since teenagers are in transition from young children to adults in physical development, their skin microbiomes are in transition as well, with a higher similarity to adults than young children, providing an explanation for the age differences in AD from a perspective of skin microbiome.

### 3.3 Microbial heterogeneity varies between skin sites in AD

It is known that different sites in human skin are colonized by distinct microbial communities (Byrd et al., 2018; Manus et al., 2020; Boxberger et al., 2021). Moreover, the microbial heterogeneity varies between skin sites in AD lesions, which may reflect disease severity. For instance, although correlations exist between species in the microbiota of nose and skin, the nose and skin harbour distinct microbial communities in pediatric AD (Totté et al., 2019). Meanwhile, the skin and oral cavity of AD patients exhibit differential reductions in microbial diversity compared with healthy individuals, which are distinctly correlated with disease severity (Li et al., 2019). Additionally, the different habitats of AD patients exhibited site-specific alterations at the genus level, with many skin-specific microbes showing opposing directions of enrichment in oral cavity (Li et al., 2019). Specifically, the relative abundance of *S. aureus* is associated with disease severity in the posterior thigh, but not in the upper back of AD patients (Ottman et al., 2021). Though it is unclear what is the cause for this distinct microbial colonization, it shows that AD may select for specific microbes, such as *S. aureus* in different anatomical locations (Ottman et al., 2021). Above researches highlight the importance of considering the variability across skin sites when studying the microbial heterogeneity in AD.

## 4 Exogenous factors that contribute to the dysbiosis of skin microbiota in AD patients

Despite the growing understanding of the close correlation between endogenous factors and AD dysbiosis, various external factors—such as personal care products (including soaps and detergents), temperature and/or climate changes, environmental triggers like air pollution, ultraviolet radiation, and allergenic foods/diet—can also disrupt the balance of skin microbiota, leading to skin dysbiosis and contributing to the exacerbation of AD.

As we all know, skin barrier disruption is one of the contributing factors that promote the development of AD (Yue et al., 2021). Consequently, irritants or environmental factors that compromise the skin’s barrier function can increase the skin’s susceptibility to microbial colonization, even resulting in skin inflammation (Yue et al., 2021). For example, factors that determine water hardness, such as high domestic water calcium carbonate concentrations, positively correlated with the prevalence of infant AD (Perkin et al., 2016). Meanwhile, the effect of mineral content in tap water were greater in children carrying FLG mutations, who favor AD development and flares (Perkin et al., 2016; Jabbar-Lopez et al., 2020). Additionally, climate changes, such as dry climates and low humidity, can exacerbate AD symptoms by disrupting the skin barrier and altering the skin microbiota (Darlenski et al., 2021; Wrześniewska et al., 2024). Moreover, personal hygiene practices or hygiene habits, such as friction and the frequent use of alkaline or antimicrobial soaps, can disrupt the skin microbiome and facilitate the pathogenesis of AD (Jing et al., 2020; Skowron et al., 2021). Furthermore, there is growing evidence that diet and gut microbiota are closely associated with skin dysbiosis and the prevalence of AD (Tham et al., 2024). A clear link between gut microbiome imbalances and AD suggests the potential influence of the gut-skin axis on skin inflammation (Moniaga et al., 2022; Hoskinson et al., 2024; Jiang et al., 2024; Rios-Carlos et al., 2024).

## 5 Topical therapies change the skin microbiota in AD patients

Therapeutics have shown great success on AD treatment in recent years. Meanwhile, different treatments may lead to varied skin microbiota shifts in AD patients (Guo et al., 2022). Herein, we summarized the recent progresses that different treatments influencing the skin microbiota alterations in AD (**Table 1**).

**Table 1:** Summary of changed microorganisms during different treatments.

Types of treatments	Study subjects and Disease severity	Study outcomes	Refs
Emollient treatment	Children/Adults with moderate AD	Emollient improved the clinical symptoms of AD patients, with the microbial communities in lesional skin more closely resembled healthy skin after treatment. It also induces beneficial mycobiome change in AD skin.	(Seite et al., 2014; Seité et al., 2017; Chandra et al., 2018; Glatz et al., 2018; Hülpüsch et al., 2020; Capone et al., 2023)
Bleach bath	Children with moderate-to-severe AD	TCS + bleach bath or TCS alone treatment normalized the bacterial compositions on lesional skin in AD. Additionally, standard treatment plus dilute bleach baths improved disease severity, with significantly lower *S. aureus* burden in moderate-to-severe AD patients.	(Gonzalez et al., 2016; Khadka et al., 2022)
Apple cider vinegar	AD patients of all grades (age ≥ 12 years)	No difference of cutaneous *S. aureus* in AD patients was observed after 2 weeks’ treatment of 0.5% apple cider vinegar.	(Luu et al., 2021)
Topical coal tar	AD patients with mean EASI of 19.8	The microbiota composition in the AD skin shifts toward the healthy skin. Meanwhile, coal tar treatment restores the antimicrobial peptide levels in AD skin.	(Smits et al., 2020)
Ozone treatment	Patients with moderate to severe AD	Ozone treatment decreases the disease severity, which is accompanied by decreased *S. aureus* colonization in AD patients. Additionally, ozone therapy helps to restore the cutaneous microbiological diversity in AD.	(Zeng et al., 2020)
Tacrolimus	NA	Tacrolimus positively affect the skin microbiota in AD, with increased commensal species after treatment.	(Wongpiyabovorn et al., 2019)
NB-UVB	Adult AD patients	NB-UVB treatment improves the disease severity and reduces the recurrence of eczema without additive effects on AD. Additionally, NB-UVB treatment increases the skin microbial diversity and S. aureus proportion in AD.	(Kwon et al., 2019; Lossius et al., 2022)
The 308 nm excimer light	Patients with moderate to severe AD	308 nm excimer light decreased the abundance of *S. aureus*, restored the skin barrier function, and improved the clinical symptom in AD patients.	(Kurosaki et al., 2020)
Climatotherapy	Patients with difficult to treat AD	DSC treatment attenuates the dysbiosis in AD patients, with several specific species dramatically affected by DSC. Meanwhile, the alpine climate treatment significantly changes the skin microbiota composition in AD patients, whereas no significant change found after moderate maritime climate treatment.	(Brandwein et al., 2019; van Mierlo et al., 2019)
Biologic treatment (Dupilumab)	Patients with moderate to severe, milder, and severe AD	The clinical improvement of AD mediated by dupilumab treatment is positively correlated with increased microbial diversity and reduced abundance of *S. aureus* on the skin.	(Callewaert et al., 2020; Lee et al., 2021; Olesen et al., 2021)

### 5.1 Topical emollient treatment changes the skin microbiota in AD patients

Topical emollients are considered as a first-line and mainstay therapy for AD treatment, which can reduce disease severity both in children and adults with AD (Tiplica et al., 2018; Pinter et al., 2019). Moreover, emollient therapy from birth represents an effective and safe approach for AD prevention (Simpson et al., 2014; Skjerven et al., 2020; Zhong et al., 2022). Further, emollients are used as monotherapy or adjuncts to soften and moisturize the skin (Hon et al., 2013; Giam et al., 2016; van Zuuren et al., 2017). However, the qualities and clinical effects of different emollients vary significantly, and only certain types are able to improve the skin’s barrier function and protect against irritants that trigger eczema (Danby et al., 2022; Elias, 2022). It was recently reported that one specific emollient associated with selected carbon material reduced the number and severity of AD flares. Concurrently, it normalized the skin microbiota compared with other emollients (Seité et al., 2017), suggesting that skin microbiota shifts could be used as biomarkers to evaluate the efficacy of different emollients during AD treatment.

Through clinical analysis and high-throughput sequencing of the skin microbiome, Seité et al. revealed that emollient improved the clinical symptoms in 72% of the AD patients, with the microbial communities in lesional skin more closely resembled unaffected skin after treatment, as indicated by increased overall diversity and decreased *Staphylococcus* species (Seite et al., 2014). Interestingly, the skin physiology, cutaneous microbiome, and AD severity were not affected by the pH of applied emollient (either pH 8.5 or pH 5.5). Instead, the skin pH tightly regulated by intrinsic factors limited the abundance of *S. aureus* in AD (Hülpüsch et al., 2020). Consistently, decreased skin pH, as well as increased *S. salivarius* population after emollient use, contribute to the preventative effects of emollient in infants at high-risk for developing AD (Glatz et al., 2018). Appropriate use of formulated emollient also increases the richness of skin microbiome, as well as the levels of ceramides and free fatty acids that play important roles in skin barrier integrity in infants, further underlining the predictive role of skin microbiota during the management of infant AD using emollient (Capone et al., 2023).

Emollient treatments also induce changes in fungal microbiome in AD, since the mycobiomes of pre-treatment and post-treatment samples cluster differently at all taxa levels by principal coordinate analysis (Chandra et al., 2018). Specifically, gram-negative *Pseudomonas* spp. significantly correlates with pathogenic fungal species (*Aspergillus*, *Candida* spp.) in lesional skins of pre-treatment group, but not in the post-treatment group (Chandra et al., 2018). Furthermore, lesional skins exhibit significant correlation between gram-positive bacteria (*Corynebacterium kroppenstedtiian* and *Staphylococcus pettenkoferi*) and pathogenic *Candida* species in the pre-treatment group, but not in the post-treatment group (Chandra et al., 2018). Thus, emollient induces beneficial changes in the skin mycobiome and modulates the microbe homeostasis in AD skin.

### 5.2 Bleach bath changes the skin microbiota compositions in AD patients

Patients with AD are prone to skin infections, most of which are suspected of contributing to pathogenesis. Thus, bleach baths could improve AD symptoms by reducing the skin microbial burden.

In a randomized, placebo-controlled, and single-blinded trial, skin samples from children with/without AD were examined at baseline and after 4 weeks’ treatment with topical corticosteroid (TCS) alone or TCS plus bleach bath. After TCS + bleach bath or TCS alone treatment, bacterial compositions on the lesional skins normalized, resembling non-lesional skin, with a tendency of restoring to the healthy skins (Gonzalez et al., 2016), indicating that TCS treatment alone is sufficient to normalize the cutaneous microbiota in AD. However, it does not mean that bleach baths have no additional impact on the skin microbiota, since 4 weeks’ time period may not be long enough for this kind of observation. Consistently, both standard treatment (emollient plus TCS) + dilute bleach bath (DBB) and only standard treatment improved disease severity in moderate-to-severe AD patients (Khadka et al., 2022). However, standard treatment with addition of DBB had significantly lower *S. aureus* burden than those who received only standard treatment over a three-month period (Khadka et al., 2022). Moreover, chronic use of DBB with intermittent application of mupirocin ointment decreased the disease severity of AD patients with clinical signs of secondary bacterial infections (Huang et al., 2009), further supporting its beneficial role of controlling bacteria load in AD therapy.

### 5.3 Additional topical treatments altering the skin microbiota in AD patients

Apple cider vinegar has been shown to have antibacterial effects. Interestingly, no significant difference in cutaneous *S. aureus* presentation was observed in AD patients before and after 2 weeks’ treatment of 0.5% apple cider vinegar (Luu et al., 2021). However, additional studies are needed to explore the effects of higher concentrations and longer period of apple cider vinegar treatment on the skin microbiota in AD.

Upon topical coal tar treatment, a useful therapeutic for atopic hand and foot eczema (van den Bogaard et al., 2013; Wollenberg et al., 2020), *Staphylococcus* abundance decreased, whereas *Propionibacterium* abundance increased in AD skins (Smits et al., 2020), suggesting a shift of the microbiota composition toward that of healthy skin. Meanwhile, coal tar treatment restored the antimicrobial peptide levels in AD skins (Smits et al., 2020), confirming that coal tar can combat AD through targeting the skin microbiota and inflammation.

Ozone treatment improves AD conditions by decreasing the disease severity (Travagli et al., 2010; Zeng and Lu, 2018), which is accompanied by decreased *S. aureus* colonization in AD lesions (Zeng et al., 2020). Additionally, ozone therapy helps to restore the cutaneous microbiological diversity in AD patients (Zeng et al., 2020), revealing its role in modulating the skin microbiota in AD.

Tacrolimus, a calcineurin inhibitor, has been widely used as a maintenance therapy in AD (Cury Martins et al., 2015; Paller et al., 2020). Interestingly, tacrolimus positively affects the skin microbiota in AD, with increased commensal species observed following treatment (Wongpiyabovorn et al., 2019), underlining a novel mechanism that tacrolimus works as an useful strategy to alleviate AD.

## 6 Phototherapy or climatotherapy influence the skin microbiota in AD patients

Phototherapy is an effective treatment in dermatology, which has long been used for the management of various inflammatory skin diseases, such as AD (Ortiz-Salvador and Pérez-Ferriols, 2017; Kemény et al., 2019; Musters et al., 2021). For instance, narrow-band Ultraviolet B (NB-UVB) is widely used in AD treatment and can significantly improve the disease severity (Lossius et al., 2021). Interestingly, a drastic increase in the skin microbial diversity and decrease in the cutaneous *S. aureus* proportion are observed in AD patients following NB-UVB treatment (Kwon et al., 2019). Coincidentally, shift towards higher diversity in the microbiota of lesional skins after NB-UVB treatment is observed in AD patients, which is associated with disease improvement (Lossius et al., 2022), confirming the role of NB-UVB in regulating skin microbiota homeostasis.

The 308 nm excimer light is another effective treatment used in AD (Nisticò et al., 2008; Oh et al., 2016). It was recently reported that the 308 nm excimer light significantly changed the bacterial composition, as well as improved the skin barrier function in the lesional skins of AD (Kurosaki et al., 2020). In addition, the treatment decreased the relative abundance of *S. aureus*, which was correlated with improved clinical symptom in AD skins (Kurosaki et al., 2020), suggesting that alterations of the skin microbiota within excimer light treatment are partially involved in the improvement of AD severity. In dogs with canine AD, the 308-nm excimer light changed the composition and diversity of the skin microbiota, with increased abundance of phyla *Actinobacteria* and *Cyanobacteria*, and decreased abundance of *S. pseudintermedius* (Park et al., 2021). More importantly, it significantly alleviated the severity of canine AD without causing any serious side effects (Park et al., 2021), indicating that excimer light is also a suitable and safe therapy for canine AD.

Climatotherapy, including Dead Sea climatotherapy (DSC), alpine and moderate maritime climates, can improve the patient’s skin condition and have been widely used for the treatment of AD (Harari et al., 2000; Adler-Cohen et al., 2012; Fieten et al., 2015; Kudish et al., 2016; Heeringa et al., 2018). Notably, DSC treatment could partially attenuate the dysbiosis in the lesional skins of AD patients (Brandwein et al., 2019). Meanwhile, severe AD skins underwent the most significant community shifts after DSC treatment. Specifically, *S. epidermidis*, *Streptococcus mitis*, and *Micrococcus luteus* were significantly affected by DSC (Brandwein et al., 2019), showing new perspectives in the climatotherapy for AD. However, not all climate therapies affect skin microbiota in AD patients. The alpine climate treatment significantly changes, whereas moderate maritime climate treatment does not change the composition of skin microbiota on the lesional skins of AD patients (van Mierlo et al., 2019).

## 7 Biologic treatment (dupilumab) changes the skin microbiota in AD patients

Dupilumab is a humanized antibody to IL-4 receptor α, which is effective in blocking IL-4 and IL-13 signaling, and reducing Th2 response (Gooderham et al., 2018). Currently, dupilumab is the only biologic medication approved by the US FDA for moderate-to-severe AD in adults and children, which can significantly improve symptoms and life quality of patients suffering from AD worldwide (Guttman-Yassky et al., 2019; Ariëns et al., 2020; Simpson et al., 2021; Zhao et al., 2022; Simpson et al., 2023). Interestingly, dupilumab rapidly reduces *S. aureus* abundance in subjects with moderate-severe AD, when compared with placebo. Importantly, patients with the greatest *S. aureus* reductions had the best clinical outcomes after dupilumab treatment (Pinter et al., 2019). Mechanistically, IL-4 receptor α blockade promotes *S. aureus* clearance partially by enhancing IL-17A expression from sites of allergic skin inflammation (Leyva-Castillo et al., 2023). The clinical improvement of AD mediated by dupilumab treatment is also positively correlated with increased microbial diversity (Callewaert et al., 2020; Olesen et al., 2021). Thus, dupilumab treatment changes the skin microbiome and ameliorates disease severity in AD. In consistent with this, recent data showed that dupilumab decreased the Eczema Area and Severity Index (EASI) as well as the load of *S. aureus* in AD patients (Lee et al., 2021). Concurrently, the microbial diversity and the abundance of *Cutibacterium* species increased (Lee et al., 2021), which were correlated with stratum corneum hydration levels and EASI improvement, suggesting that Th2 blockade-induced skin microbiotal normalization is associated with improved skin barrier properties in AD. Moreover, systemic dupilumab treatment tends to shift the skin microbiome of patients with moderate-to-severe AD toward a healthy skin flora, which is largely independent of the clinical response, indicating its direct regulation on the skin microbiome (Hartmann et al., 2023).

## 8 The implication of skin microbiota in AD treatment

Alteration of the skin microbiota, especially the *S. aureus* colonization, is closely associated with disease severity in AD (Hendricks et al., 2019). Accordingly, commensal skin bacterium decolonizating *S. aureus* holds therapeutic benefits for AD patients (**Table 2**).

**Table 2:** Representative microbiota-based therapies applied in AD treatment.

Names of bacteria applied	Experimental models/Clinical studies	Outcomes/Results	Refs
*Roseomonas mucosa* (*R. mucosa*)	Mouse model of AD, open-labeled trial, and randomized and placebo-controlled trial	*R. mucosa* from healthy people improved outcomes in mouse model of AD. Clinically, *R. Mucosa* significantly decreased *S. aureus* burden and disease severity in both adult and pediatric patients, with few/no adverse events	(Myles et al., 2016; Myles et al., 2018; Myles et al., 2020; Liu et al., 2022)
Autologous antimicrobial-producing CoNS (CoNS-AM^+^)	Double-blind, vehicle-controlled and single-center randomized clinical trial	CoNS-AM^+^ treatment improved clinical outcomes and reduced *S. aureus* colonization on lesional skin of AD patients, with no serious adverse events observed	(Nakatsuji et al., 2021a)
*Staphylococcus hominis A9* (*ShA9*)	AD mouse model and a phase 1, double-blinded, randomized trial	*ShA9* killed *S. aureus* and inhibited PSMa expression in the mouse model. Meanwhile, *ShA9* improved the disease severity, decreased the *S. aureus* burden with few adverse events in AD patients	(Nakatsuji et al., 2021b)
*S. epidermidis*	Agar well diffusion assay, *in vitro* model	Live planktonic *S. epidermidis* inhibited *S. aureus* growth. Additionally, thermolabile cytoplasmic bacteriocin extracted from *S. epidermidis* exhibited selectively antimicrobial activity against *S. aureus* and methicillin-resistance *S. aureus*	(Nakatsuji et al., 2017; Jang et al., 2020)

### 8.1 Topical R. mucosa transplantation is a safe and potent therapy in AD treatment

The culturable gram-negative bacteria from healthy people but not from AD patients are associated with enhanced barrier function, activated immunity, and controlled *S. aureus* colonization (Myles et al., 2016). Particularly, one commensal, *Roseomonas mucosa* (*R. mucosa*) from healthy skins, improves disease severity of AD in a mouse model, suggesting its therapeutic potential for AD treatment (Myles et al., 2016). Recently, a first-in-human topical microbiota transplantation using *R. mucosa* was conducted for AD treatment. In the open-labeled trial, AD patients topically treated with *R. mucosa* showed decreased *S. aureus* burden and disease severity, with no adverse events (Myles et al., 2018), supporting the efficacy and safety of *R. mucosa* therapy in AD treatment. More recently, a randomized and placebo-controlled trial of *R. mucosa* treatment in children with AD was conducted. In this trial, *R. mucosa* ameliorated disease severity, improved skin barrier function, decreased *S. aureus* abundance on the skin, and reduced topical steroid use without severe adverse events (Myles et al., 2020), further confirming that topical *R. mucosa* transplantation in AD patients is warranted.

To enhance the effects of *R. mucosa* transplantation long-termly, a living bacterial formulation that integrates *R. mucosa* with poly (vinyl pyrrolidone), poly (vinyl alcohol), and sodium alginate into a skin dressing was developed (Liu et al., 2022). Functionally, the skin dressing recovers skin barrier functions and alleviates AD-associated inflammation. Meanwhile, it serves as extrinsic culture harbors and nutrient suppliers to support *R. mucosa* survival (Liu et al., 2022), offering a clue that the combination of topical bacteria transplant with biomaterials represents an effective bacteriotherapy toward AD treatment.

### 8.2 CoNS transplantation represents a potential herapy in AD treatment

As outlined in “2.2 Section”, the skin commensals, coagulase-negative staphylococci (CoNS) species residing on normal skins, inhibit *S. aureus* growth and development of AD (Nakatsuji et al., 2017; Paharik et al., 2017; Williams et al., 2019). Interestingly, *S. aureus* burden on the lesional skins of patients who received autologous antimicrobial-producing CoNS (CoNS-AM^+^) treatment was reduced by 99.2% compared with vehicle treatment, in a double-blind, vehicle-controlled, and single-center randomized trial (Nakatsuji et al., 2021a). Meanwhile, no serious adverse events were observed after treatment. Importantly, the autologous CoNS-AM^+^ treatment improved clinical outcomes in AD patients (Nakatsuji et al., 2021a), suggesting that autologous CoNS strains can safely decrease *S. aureus* colonization and improve disease severity.

*Staphylococcus hominis* A9 (ShA9), a bacterium isolated from healthy human skins, kills *S. aureus* on the skin of mice and inhibits PSMa expression from *S. aureus* (Nakatsuji et al., 2017; Nakatsuji et al., 2021b). Additionally, a first-in-human trial of topical ShA9 on the skins of adults with *S. aureus*-positive AD was conducted. The topical ShA9 treatment showed few adverse events on participants, while a significant decrease in *S. aureus* was observed after ShA9 treatment (Nakatsuji et al., 2021b). Although some *S. aureus* strains on participants were not directly killed by ShA9, mRNA expression of PSMa was inhibited in all strains. The *post-hoc* analysis revealed improvement in disease severity by ShA9 (Nakatsuji et al., 2021b), demonstrating the benefits of ShA9 bacteriotherapy for AD treatment.

*S. epidermidis* is a well-known commensal bacteria that selectively kills *S. aureus* (Nakatsuji et al., 2017), suggesting its protective role against AD. On the agar well diffusion assay, live planktonic *S. epidermidis* clearly inhibits *S. aureus* growth, but heat-killed cells do not show this effect (Jang et al., 2020), leading to the hypothesis that cytoplasmic bacteriocin from *S. epidermidis* could be a promising agent to inhibit *S. aureus* growth. In fact, a novel thermolabile cytoplasmic bacteriocin extracted from *S. epidermidis* exhibits selectively antimicrobial activity against *S. aureus* and methicillin-resistance *S. aureus* (Jang et al., 2020). However, more data should be obtained to confirm its potency and safety, before its clinical use for AD treatment.

### 8.3 Additional products targeting the skin microbiota in AD treatment

Antibiotics specific for bacterial species including *C. mastitidis*, *S. aureus*, and *C. bovis*, almost completely reverse dysbiosis and eliminate the skin inflammation in mouse models (Kobayashi et al., 2015). However, it should be concerned that the use of antibiotics may trigger antibiotic resistance and more serious dysbiosis. ATx201 (niclosamide, a small molecule), was recently reported to reduce both *S. aureus* and methicillin-resistant *S. aureus* colonization, without affecting the skin commensals (Weiss et al., 2022). Additionally, in a phase 2 trial, ATx201 ointment effectively reduced *S. aureus* colonization and increased the Shannon diversity of skin microbiomes in patients with mild-to-severe AD (Weiss et al., 2022), revealing that small molecules could work as a decolonizing agent in AD treatment.

Probiotics are potent immune-modulators used in AD management (Dissanayake and Shimojo, 2016; Fanfaret et al., 2021; Herbert et al., 2023). A cosmetic lotion containing heat-treated Lactobacillus johnsonii NCC 533, was recently demonstrated to control *S. aureus* colonization as well as improve clinical outcomes in AD patients, supporting the development of topical lotion containing probiotics for AD treatments (Blanchet-Réthoré et al., 2017). More recently, ointment containing live Lactobacillus reuteri DSM 17938 was also shown to improve the local symptoms in adult AD patients (Butler et al., 2020). Most importantly, Lactobacillus reuteri DSM 17938 is cutaneously acceptable and safe, confirming that probiotics are safe and promising therapeutics in the management of AD.

Natural products represent new therapeutics combating against AD. Aquaphilus dolomiae (ADE) is an aqueous protein extract, which increases IL-10 expression in monocyte-derived dendritic cells (moDC) (Martin et al., 2016). Meanwhile, ADE-moDC counteracts the proliferative effect and mitogenic effect of *S. aureus* on CD4^+^ T cells (Martin et al., 2016). Thus, owing to the role of *S. aureus* in driving inflammation in AD, the immunosuppressive property of the ADE might be useful to reduce disease severity. Moreover, topical application of a *Castanea sativa* extract prevents the progression of AD, through increasing filaggrin, claudin-1, and loricrin expression, while decreasing *S. aureus* virulence (Cadau et al., 2022), further confirming that natural products hold great potential for AD treatment.

## 9 Prospects and perspectives

The dysbiosis of skin microbiota is widely accepted as important mechanisms in the pathogenesis of AD. Specifically, the decreased microbial diversity and increased *S. aureus* colonization are closed associated with disease severity in AD. Meanwhile, therapeutics targeting the cutaneous microbiota represent a novel strategy for AD treatment. Despite the significant potential of microbiota-based therapies for AD, the treatment of dysbiosis should be integrated with standard skincare practices, since AD is a complex skin disorder that requires a multifaceted approach, incorporating various skincare modalities and treatments for effective management. Although numerous studies, even several early phage clinical trials have shown that skin microbiota-based therapeutics hold great promise for the treatment of AD, the precise mechanisms remain to be elucidated, and many problems remain to be solved for their better clinical applications. For instance, current studies mainly focus on the role of skin microbiome in AD pathogenesis and its potential use in AD treatment. However, we cannot ignore that changes and dysregulations of skin microbiome can work as biomarkers for AD diagnosis, especially now that a variety of non-invasive sampling methods are developed (Elpa et al., 2021; Andersson et al., 2022; Shima et al., 2022). Meanwhile, we need to build/construct a systematic model, which comprehensively considers the skin microbiota and host as a whole that involved in the management of AD, since skin microbiota is affected by multiple internal and external factors during AD pathogenesis. The current researches regarding the interaction between skin microbiota and AD were mainly conducted on cellular or mouse models, some were conducted on preclinical or phase I/II clinical models, thus there is still a long way to go before these investigations can be truly applied to the clinic. However, we are confident about the application prospects of skin microbiota-based therapeutics and believe that theses therapeutics can be widely promoted for AD treatment in the near future.
